# Impaired Functional Connectivity Underlies Fragile X Syndrome

**DOI:** 10.3390/ijms23042048

**Published:** 2022-02-12

**Authors:** Lital Gildin, Rossana Rauti, Ofir Vardi, Liron Kuznitsov-Yanovsky, Ben M. Maoz, Menahem Segal, Dalit Ben-Yosef

**Affiliations:** 1 Wolfe PGD Stem Cell Lab, Racine IVF Unit, Lis Maternity Hospital Tel-Aviv Sourasky Medical Center, Tel-Aviv 64239, Israel; litalovadia@mail.tau.ac.il (L.G.); lirony1@mail.tau.ac.il (L.K.-Y.); 2 Department of Cell and Developmental Biology, Sackler Faculty of Medicine, Tel-Aviv University, Tel-Aviv 69978, Israel; 3 Department of Biomedical Engineering, Tel-Aviv University, Tel-Aviv 69978, Israel; rossanarauti@mail.tau.ac.il (R.R.); ofirvardi1234@gmail.com (O.V.); bmaoz@tauex.tau.ac.il (B.M.M.); 4 School of Electrical Engineering, Tel-Aviv University, Tel-Aviv 69978, Israel; 5 Sagol School of Neuroscience, Tel-Aviv University, Tel-Aviv 69978, Israel; 6 The Center for Nanoscience and Nanotechnology, Tel-Aviv University, Tel-Aviv 69978, Israel; 7 Department of Brain Sciences, The Weizmann Institute, Rehovot 76100, Israel

**Keywords:** Fragile X syndrome, disease modeling, human embryonic stem cells, neural differentiation, MEA, electrophysiology

## Abstract

Fragile X syndrome (FXS), the most common form of inherited intellectual disability, is caused by a developmentally regulated silencing of the *FMR1* gene, but its effect on human neuronal network development and function is not fully understood. Here, we isolated isogenic human embryonic stem cell (hESC) subclones—one with a full FX mutation and one that is free of the mutation (control) but shares the same genetic background—differentiated them into induced neurons (iNs) by forced expression of *NEUROG-1*, and compared the functional properties of the derived neuronal networks. High-throughput image analysis demonstrates that FX-iNs have significantly smaller cell bodies and reduced arborizations than the control. Both FX- and control-neurons can discharge repetitive action potentials, and FX neuronal networks are also able to generate spontaneous excitatory synaptic currents with slight differences from the control, demonstrating that iNs generate more mature neuronal networks than the previously used protocols. MEA analysis demonstrated that FX networks are hyperexcitable with significantly higher spontaneous burst-firing activity compared to the control. Most importantly, cross-correlation analysis enabled quantification of network connectivity to demonstrate that the FX neuronal networks are significantly less synchronous than the control, which can explain the origin of the development of intellectual dysfunction associated with FXS.

## 1. Introduction

Fragile X syndrome (FXS) is the most prevalent form of inherited intellectual disability. FXS patients present a spectrum of developmental and behavioral problems including learning disabilities and cognitive impairment. The disease results from a developmentally regulated silencing of the Fragile X (FX) Mental Retardation Protein (FMRP) [1], an RNA-binding protein essential for proper synaptic architecture and plasticity [2]. Research models of FX include human neural progenitor cells taken from aborted fetuses [3,4] and *FMR1* knockout (KO) animals that share some phenotypes with humans [5,6,7]. The FX phenotype is associated with several cellular defects including abnormal dendritic spine morphology, nonsense-mediated mRNA decay and altered intrinsic neuronal properties [8,9,10,11]. However, there are substantial differences between the human brain and that of the mouse model [12]; moreover, recent clinical trials in FXS patients, based on animal models, failed to ameliorate disease-related symptoms [13,14,15], highlighting the difficulty in translating findings directly from animal models to human patients.

Human pluripotent stem cells (hPSC) have emerged as a powerful tool to investigate the pathogenesis of neurological diseases, due to their ability to differentiate into neural cells in vitro. We have previously differentiated several FX-human embryonic stem cell (hESC) lines into functional neurons with inactivated *FMR1*, demonstrating that they can serve as a good research model for exploring the molecular and functional mechanisms underlying FXS [16]. However, differentiation of FX-PSCs led to poor neuronal maturation and decreased spike frequencies relative to control neurons [16,17]. Nevertheless, other studies demonstrated inconsistent results regarding the functional differences between FX-PSCs and control neurons [18,19,20]. In the current study, hESC isogenic subclones were generated, one with a full FX mutation and one that is free of the mutation (WT) but shares the same genetic background, enabling us to overcome variability between lines. These isogenic subclones were directly converted into induced neurons (iNs) with mature intrinsic and synaptic properties, by ectopic expression of the neuronal transcription factors neurogenin-1 (*NEUROG-1*). This technology allows for more efficient, rapid and reproducible production of neurons that form synapses [21,22] enabling us to model the neuronal network functions of diseased cells in an in vitro system. The high-throughput Multi-Electrode Array (MEA) allows us to show that, apart from the FX neuronal networks being hyperexcitable, they also have higher spontaneous burst-firing activity. Most importantly, we were able to quantify neuronal network activity and demonstrate that the FX networks are significantly less synchronous than controls, which can explain the origin of the development of intellectual dysfunction associated with FXS. Our robust platform, based on neural differentiation of isogenic FX-hESC lines combined with functional analysis, enabled us to explore the pathophysiological basis of FXS, and will potentially serve as a more reliable human preclinical drug screening model.

## 2. Results

### 2.1. Generation of Isogenic FXS Full-Mutation hESC Subclones

**Figure 1:**
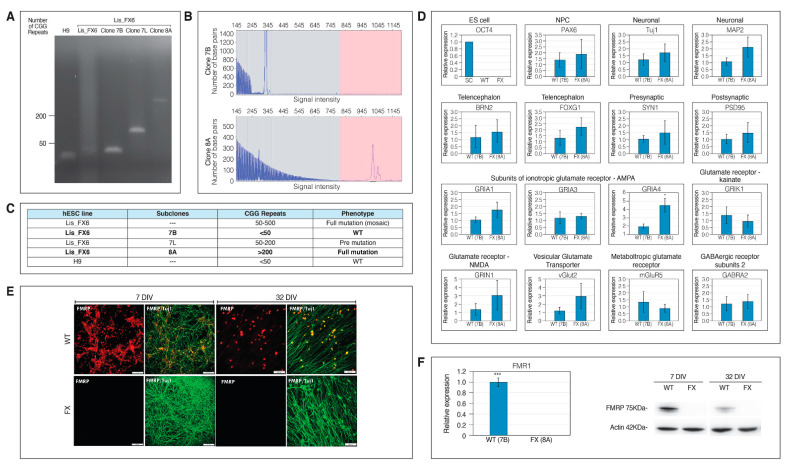
Generation of Fragile X (FX) human embryonic stem cell (hESC) isogenic clones: (**A**) Analysis of the FX-hESC isogenic clones’ CGG repeat size by PCR-based repeat-number assay of hESC. H9 (WiCell, University of Wisconsin) served as control; (**B**) AmplideX PCR/CE *FMR1* Reagents (Asuragen) commonly used for clinical diagnosis of FXS; (**C**) FX mutation status of isogenic iN clones; (**D**) neuronal characterization of iNs. Expression of neuronal genes in FX- and control-iNs. Quantitative RT-PCR analysis was performed on RNA extracted at 39 days in vitro (DIV). The housekeeping gene *GUSB* served as an internal control. Three independent experiments were performed, and data are represented as mean ± SEM. *T*-test: * *p* < 0.05 (**E**) FMRP expression in FX and isogenic control hESC 7 & 32 DIV. β-III tubulin (*Tuj1*; green) and FMRP (red). Scale bar: 200 µm; (**F**) RNA expression of *FMR1* analyzed by qRT-PCR at 39 DIV (left). Data are represented as mean ± SEM. Three independent experiments were performed. *T*-test: *** *p* < 0.001. FMRP expression using western blot analysis (right). Protein was extracted at either day 7 or at 32 DIV. Human FMRP antibody is shown, and actin served as a positive control.

**Scheme 1:**
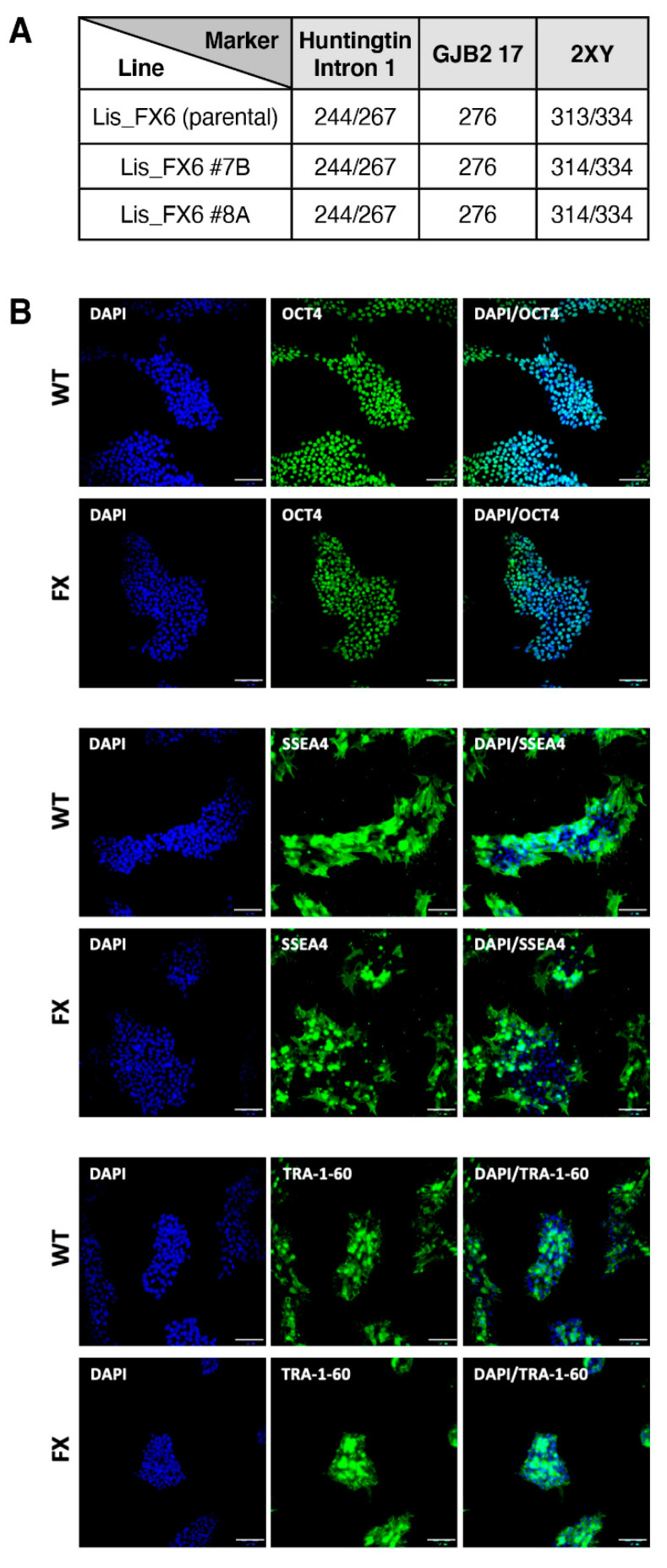

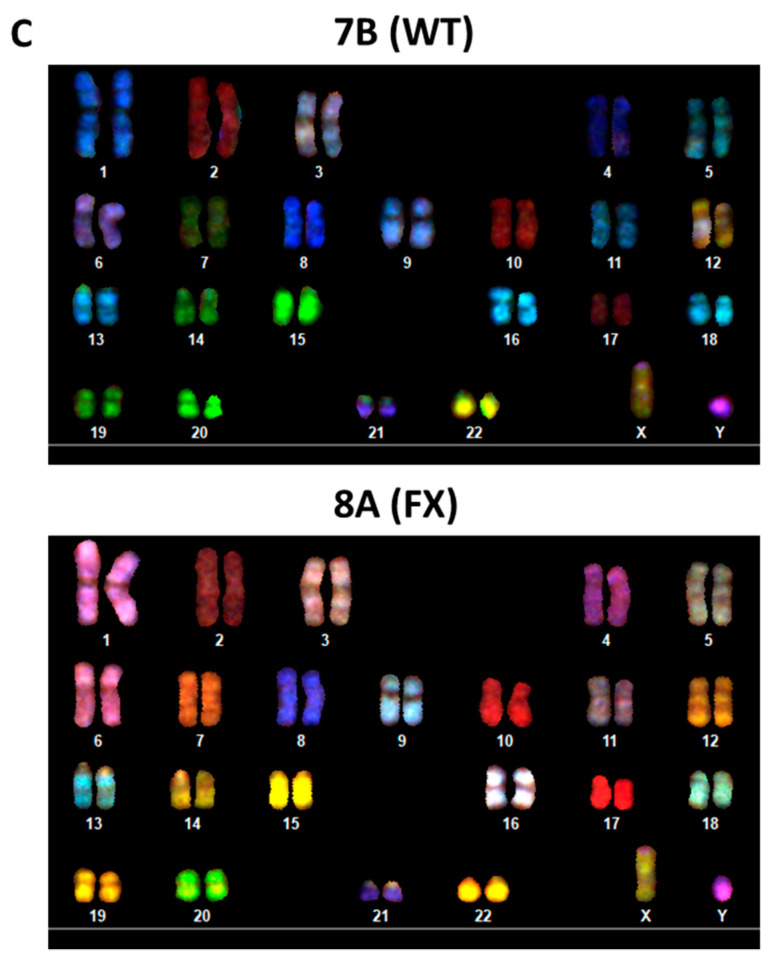
Characterization of hESC subclones generated from Lis_FX6: (**A**) Polymorphic marker analyses of CA repeats (marker), known to identify each unique hESC line, are listed. Lis_FX6 original hESC line; #—subclone derived from the original hESC line; (**B**) expression of the OCT4/ SSEA4/ TRA-160 pluripotent genes (green) in the two isogenic clones. Nuclear staining: DAPI (blue). Scale bar: 100 µm; (**C**) karyotype by SKY analysis of clone 7B at passage 48 and clone 8A at passage 46.

In previous studies, several FXS-hESC lines representing variability in FX populations were compared to several control lines to explore the role of *FMR1* in FX [23,24]. Other studies use CRISPR/Cas9 for removal of the CGG repeats or the hypermethylation of the CGG expansion mutation [19,25], but such genetic intervention is commonly associated with off-target effects. To overcome this limitation, in the current study, isogenic subclones were isolated, freed from any genetic intervention, from the Lis_FX6 hESC line that was previously derived in our lab, following PGD and present CGG repeats from the normal-through the full-mutation range. Sixty-one single-cell colonies were manually isolated and screened for their CGG repeat number in the 5’-untranslated region of *FMR1* (Figure 1A) and by the AmplideX PCR/CE *FMR1* Reagents (Asuragen, TX, USA) commonly used for clinical diagnosis of FXS (Figure 1B). Our results show that we successfully isolated an FXS subclone that is in the full mutation range (>200 CGG repeats; clone 8A), a subclone that is in the pre-mutation range (50–200 CGG repeats; clone 7L) and another subclone that is free of the mutation (<50 CGG repeats; clone 7B) that will serve in all further experiments as an isogenic control (WT; Figure 1C). These subclones were analyzed for CGG repeat length three times during the study period and the results demonstrate their stability over extended cell divisions. Importantly, all lines and subclones were shown to be pluripotent with a normal karyotype (see Scheme 1) and maintained their repeats number along the differentiation process. Induced differentiation into neurons by overexpression of *NEUROG-1* already generated neurons with typical morphology within 7 days (Figure 1E). These iNs already completely inactivated the *FMR1* gene, only in the FX full-mutation subclone, at 7 days following neuronal induction, at both the RNA and protein level (Figure 1E,F), mimicking *FMR1* silencing in FXS developing fetuses. We then characterized the expression profile of the FX-iNs and their isogenic counterparts. Our results show that by day 39, both FX- and control-iNs completely inactivated the pluripotent gene *OCT4*, and positively expressed the neural genes *PAX6*, *Tuj1* and *MAP2*. They also expressed *BRN2* and *FOXG1*, which characterize excitatory cortical neurons. The pre- and post-synaptic genes *SYN1* and *PSD-95* were also expressed in both FX and WT iNs at day 39, demonstrating their maturity. Expression analysis of the glutamate receptors showed that two of four subunits of the AMPA receptor (*GRIA1, GRIA3*) were found to be expressed in both FX and WT iNs at the same level. Interestingly, *GRIA4* expression was significantly higher in FXS neurons compared to WT (*p* < 0.05). Both FX and WT iNs also express the ionotropic glutamate receptors kainate 1 (*GRIK1*), the NMDA receptor subunit 1 (*GRIN1*), the metabotropic glutamate receptor 5 (mGluR5), and the vesicular glutamate transporter-2 (vGlut2). The GABA receptor subunit α-2 (*GABRA2*) was also expressed in these neurons, although with lower expression levels compared with glutamatergic genes (Figure 1D). Collectively, the expression of this series of neuronal markers shows that FX and WT iNs are excitatory glutamatergic neurons.

### 2.2. The Effect of FMRP Expression on Neuronal Function and Network Development

**Figure 2:**
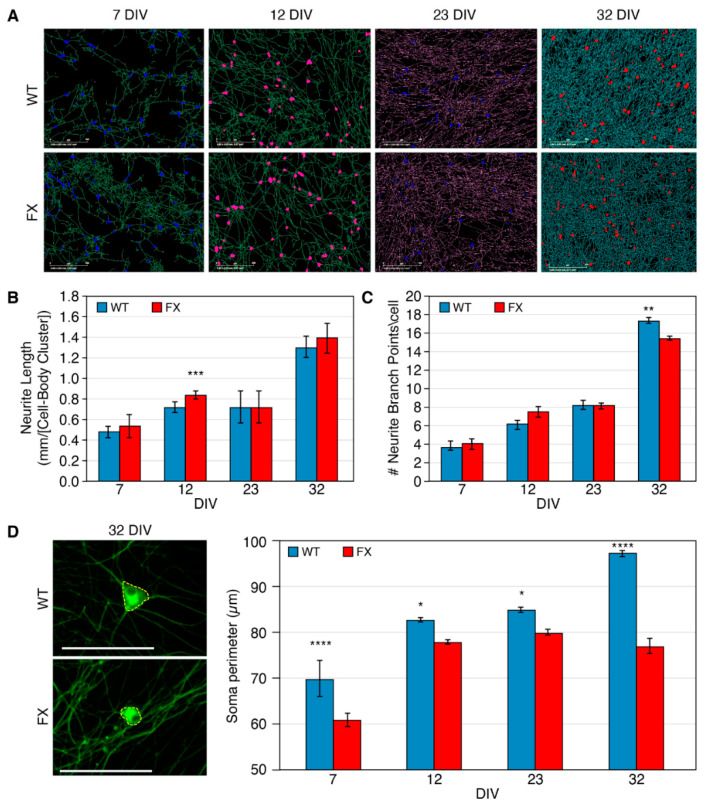
High-throughput image analysis of Fragile X (FX) induced neurons (iNs): (**A**) Representative images of WT and FX-iNs following Incucyte live-cell analysis, at four time points following neuronal induction (7, 12, 23, 32 DIV). Soma and neurites are presented in different colors. Scale bar: 200 µm; (**B**) total neurite length in mm/cell body cluster; (**C**) neurite branch points/cell body cluster. Two–three independent experiments were performed including 2500–4000 neurons at each time point. Data are represented as mean ± SEM. One-way ANOVA: ** *p* < 0.005, *** *p* < 0.0005; (**D**) representative images of iNs’ soma perimeter labeled with *Tuj1* (green), at 32 DIV. Scale bar: 50 µm (left). Quantification of soma perimeter (µm) was analyzed by the cellSens Dimension software (right). Two (23 & 32 DIV) or three (7 & 12 DIV) independent experiments were performed on each day for both neurite length and neurite branch points including 2000–4000 neurons at each time point. Data are represented as mean ± SEM. One-way ANOVA: * *p* < 0.01, **** *p* < 0.0001.

The formation and maintenance of synaptic networks are necessary for neuronal functions through continuous changes in the neurons’ fine structure. We therefore analyzed the morphology of the FXS-iNs at four time points following neuronal induction (7, 12, 23 and 32 DIV). The results show that while iNs mature in vitro, the neurites elongate gradually from day 7 to 32 (Figure 2). Temporarily increased neurite growth was detected in FX-iNs at day 12 DIV (*p* < 0.0005), compared with WT, but their length was not different from WT later during development, indicating that their delayed elongation catches up later (Figure 2B). In addition, neurite branching also increased as the iNs matured, in both FX and WT neurons. However, at day 32 of differentiation, the FX-iNs had significantly fewer branch points than the WT (15.5 and 17.5 branch points/cell body cluster, respectively; *p* < 0.005), probably reflecting their retarded maturity (Figure 2C). In addition, the soma perimeter of both FX and WT iNs increased during development, with FX-iNs presenting a significantly smaller soma at all four time points (Figure 2D). Collectively these results indicate that the lack of FMRP alters the maturation of neurons, resulting in a smaller neuronal soma and less-branched neurites.

**Figure 3:**
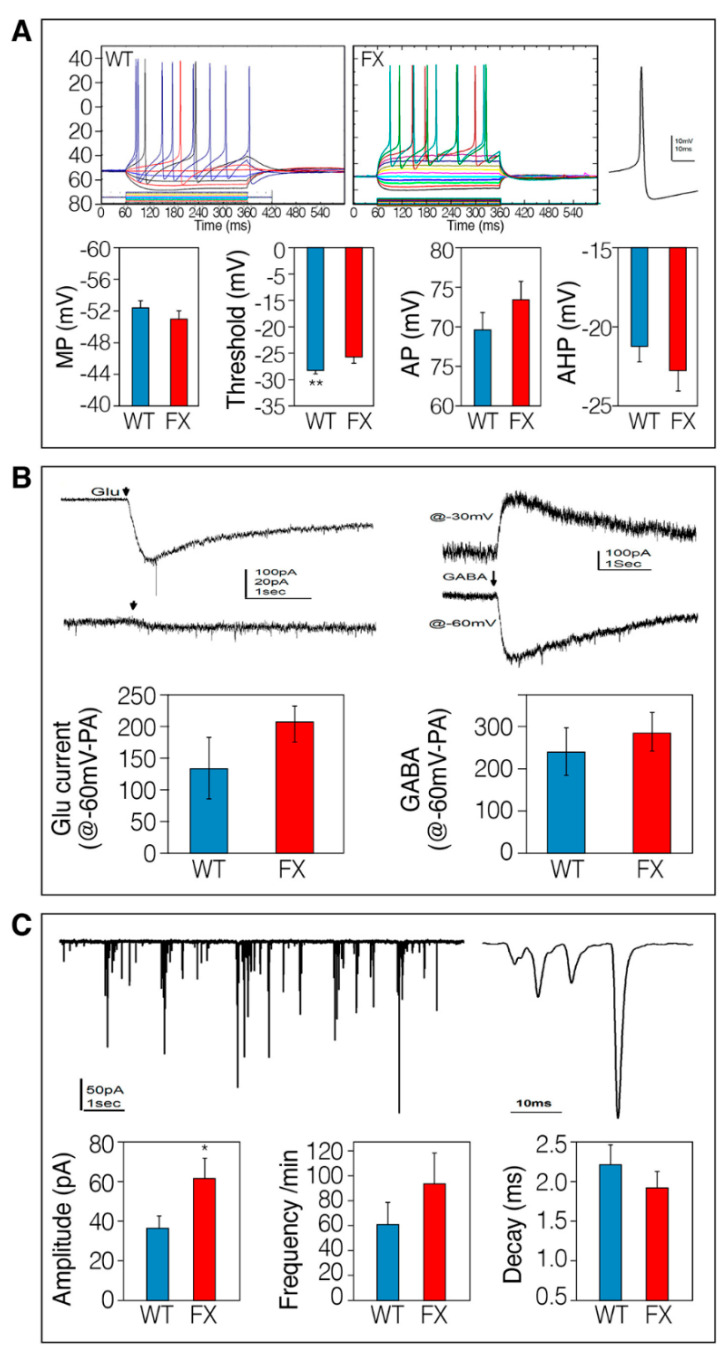
Patch-clamp recordings of Fragile X (FX)-compared to control-induced neurons (iNs): (**A**) Top: Intrinsic properties of action potentials (APs) of FX-iNs compared to WT. Sample illustration of an IV curve, depicting changes in membrane voltage in response to hyperpolarizing and depolarizing current pulses. The right is an expanded AP taken from the battery on the left. Bottom: summary of recorded neuron properties: resting membrane potential (MP); firing threshold (** *p* < 0.01); AP amplitude; after-hyperpolarization (AHP). WT: *n* = 30, and FX-iNs: *n* = 20; (**B**) iNs Glutamate and GABA responses. Responses to pressure application of glutamate onto FX-iNs at resting membrane potential (−60 mV) in voltage-clamp (left). Bottom: comparison of FX vs. WT-iNs’ response to glutamate at >40 DIV (WT, *n* = 15; FX-iNs, *n* = 10). Right: responses to GABA at resting potential (−60 mV) and at a depolarizing potential (−30 mV). Bottom: Comparison of FX vs. WT-iNs’ response to GABA at >40 DIV (WT, *n* = 7; FX-iNs, *n* = 8); (**C**) top: Spontaneous excitatory synaptic currents in FX-iNs compared to WT. Sample illustration of a trace recorded from FX neurons shown at low (left) and high (right) resolutions. Bottom: Comparison of the mean amplitude (size in pA) of synaptic current produced by FX- and control-iNs; *p* < 0.02. Frequency of spontaneous EPSC in FX- and control-neurons. The decay time of individual EPSCs. Comparisons were made between voltage-clamped (*n* = 33) WT neurons and FX-iNs (*n* = 21), held at −60 mV.

Next, we explored the functional correlates of these morphological features at the single-cell level. Basic functional properties of iNs were analyzed using the patch-clamp technique at >40 DIV. As indicated in Figure 3, the basic properties of FX-iNs were not different from WT; both can discharge repetitive action potentials (APs) each followed by a large afterhyperpolarization (AHP; Figure 3A). In addition, the resting membrane potential and the APs’ amplitude were not different between FX and WT neurons. AHPs are slightly, but not significantly, larger in the FX-iNs compared to WT neurons. Interestingly, the firing threshold of WT neurons was significantly lower than that of the FX-iNs (−27 mV compared to −24 mV, *p* < 0.01; Figure 3A). FX-iNs, similar to WT, responded to both glutamate and GABA applied by pressure from an adjacent micropipette (Figure 3B). More importantly, FX neuronal networks were able to generate slightly more spontaneous excitatory postsynaptic currents (EPSC) than WT neurons (see Figure 3C). The mean amplitude of the spontaneous synaptic currents was significantly larger in the FX-iNs (−36.4 ± 6.16 pA in controls compared to −61.2 ± 10.63 pA, *p* < 0.02), which can indicate a higher number of synapses in the FX neurons. The frequency of EPSC was slightly, but not significantly, higher in the FX neurons compared to the controls. The averaged decay time of individual EPSC was similar in both WT and FX (Figure 3C). These results indicate that even though the somatic perimeter is larger in the control than in the FX (Figure 2D), the synaptic currents, likely to reside on dendrites, are not different between the two groups. Tentatively, there was no apparent difference in capacitance between neurons in the two groups (not shown). Consequently, the APs’ initiation difference (Figure 3A) probably compensates for the higher excitability associated with an increase in synaptic activity in the FX neurons.

**Figure 4:**
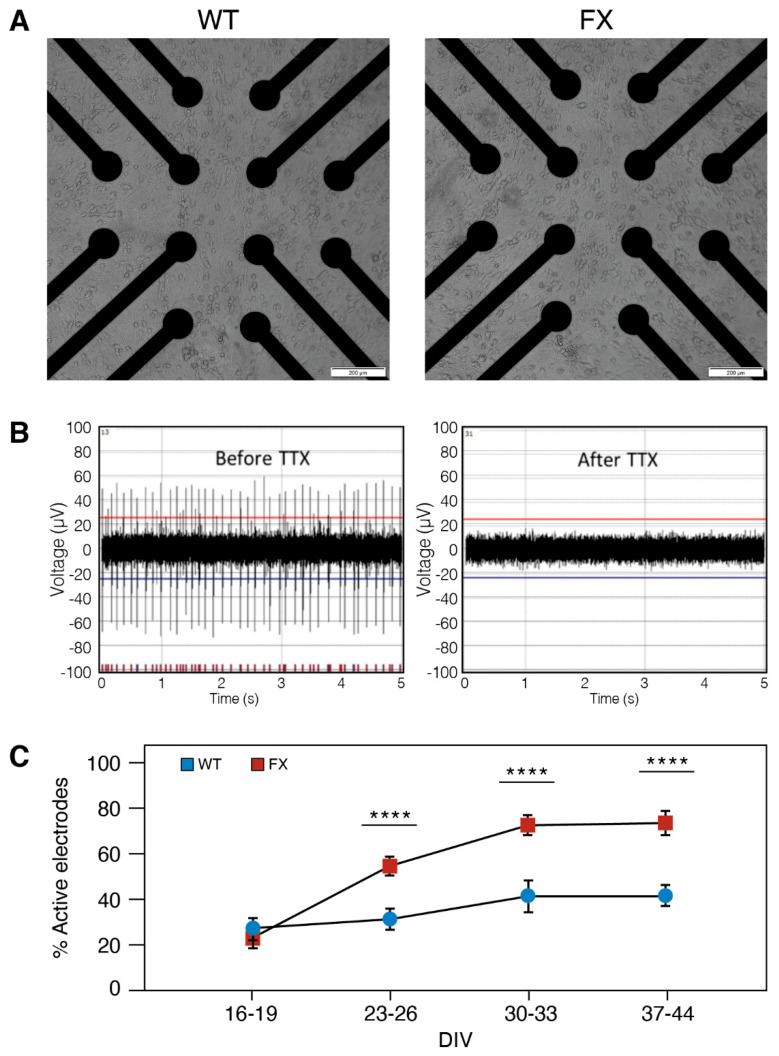
Spontaneous activity of induced neurons (iNs) recorded with MEA: (**A**) High-throughput MEA for recording spontaneous activity (24-well plate; 12 electrodes/well) with representative images of WT and FX iNs. Note the similar distribution of iNs at 10 days post induction captured from the center of a single MEA well. Scale bar: 100 µm. (**B**) representative traces of WT-iNs at 51 DIV (left) with spikes totally suppressed by the application of the Na^+^ channel blocker Tetrodotoxin (TTX; 0.5 µM; right). The red (top) and blue (bottom) lines are the thresholds (4.5 times the standard deviation of the noise); (**C**) percentage of active electrodes at four time points post-neuronal-induction (16–19, 23–26, 30–33, 37–44 DIV). The same batch of neurons was recorded for all time points. *n* = 22–36 wells were sampled for each line at each time point. Data are represented as mean ± SEM. One-way ANOVA: **** *p* < 0.0001.

MEA is a complementary approach to patch-clamp and is a gold standard for characterizing the extracellular potential of neurons and assessing the neuronal network activity in vitro. We used high-throughput MEAs which were recorded in 24-well-plates (12 electrodes/well) to compare the neural network activity of FX and WT neurons plated at the same density (10^5^ neurons/well) and conditions (Figure 4A; for details, see in Methods: MEA recordings and data analysis). Blockage of APs by Tetrodotoxin (TTX; 0.5 µM) indicates that APs in both FXS and WT neurons results from the activation of Na^+^ currents (Figure 4B). Neurons were recorded at eight different time points during differentiation (16–44 DIV), and spontaneous APs were detected using a voltage-threshold-based algorithm (Figure 4C). Approximately 60% of electrodes were active in both lines, indicating sustained network activity in both FX and WT neurons. The increase in the number of active electrodes with age in culture may represent neuronal maturation within the duration of the experiment, reaching an optimal activity of >65% of all electrodes on >30 DIV. Electrode recording from FX neurons showed a nearly two-fold higher activity (73%) compared to WT (42%) (>23 DIV; *p* < 0.0001), and the results were confirmed with the high number of recorded neurons (Figure 4C).

**Figure 5:**
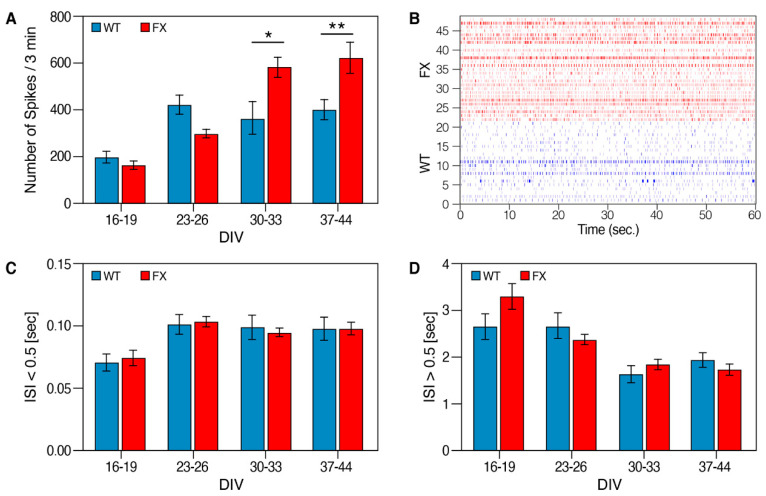
Neuronal hyperexcitability in FX-iNs: (**A**) Number of spikes per active electrode. Active electrodes were defined by presenting ≥15 spikes/minute; (**B**) representative raster plot of spikes from more than 20 electrodes at 37 DIV FX and WT-iNs, showing the pattern and density of spikes during 60 s recording. In all graphs, the voltage-threshold-based algorithm runs over 20000 Hz high-pass-filtered traces, and is adjusted to 4.5 times the standard deviation of the noise; (**C**) inter-spike interval (ISI). Average ISI below 0.5 s and (**D**) above 0.5 s. Three independent experiments were performed. The same batch of neurons was recorded for all time points. *n* = 20–48 wells were sampled for each line at each time point. Data are represented as mean ± SEM. One-way ANOVA: * *p* < 0.05, ** *p* < 0.01.

For further analysis, we included only electrodes that discharged ≥15 spikes/minute. Collectively, the FX-iNs were hyperexcitable compared to the control as seen by the significantly higher number of spikes per active electrode in FX neurons (at 30–44 DIV; Figure 5A,B). Inter-spike interval (ISI) analysis demonstrated no differences in ISI between the two lines (Figure 5C,D), probably due to the variable spike frequencies in different neurons constructing both FX and WT neuronal networks.

**Figure 6:**
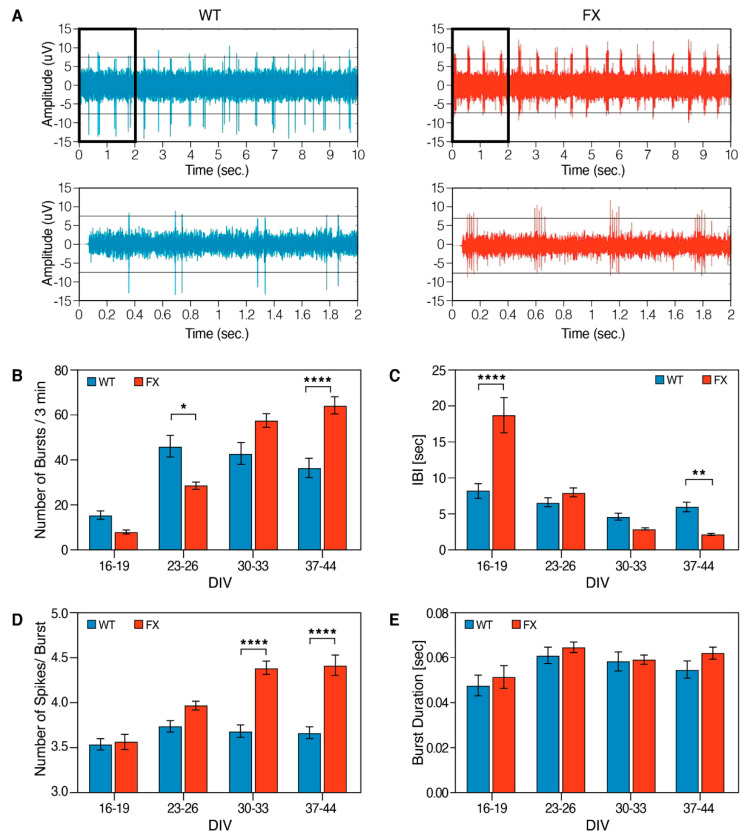
Higher spontaneous burst activity in FX neurons: (**A**) representatives burst traces of WT (blue) and FX (red) at 37 DIV during 10 s recording (top) and higher resolution of the first 2 s from each trace (bottom); (**B**) number of bursts; (**C**) intra-burst interval (IBI); (**D**) number of spikes in a burst; (**E**) burst duration. Bursts were defined as >3 spikes with <300 ms inter-spike interval. Three independent experiments were performed at each time point (16–19, 23–26, 30–33 and 37–44 DIV). *n* = 50–235 electrodes were sampled for each line in each time point. Data are represented as mean ± SEM. One-way ANOVA: * *p* < 0.05, ** *p* < 0.01, **** *p* < 0.0001.

Since neurons in the network tended to fire in bursts, we analyzed and compared the burst-firing activity between the FXS and WT neurons (Figure 6). The average rate of burst discharges was significantly higher in FXS-iNs compared to WT (64 vs. 36 bursts/ 3 min, respectively, at 37–44 DIV, *p* < 0.0001; Figure 6A,B). These results are correlated with the inter-burst interval (IBI) being decreased in mature FXS neurons (>37 DIV). Interestingly, at an earlier stage of differentiation (16–19 DIV), the results were the opposite, with an increased IBI observed in FX neurons compared to WT, probably reflecting their delayed development and maturation (Figure 6C). In accordance with the above, the averaged number of spikes in a burst was also significantly higher in the FXS-iNs compared to WT (>30 DIV; *p* < 0.0001; Figure 6D). The duration of bursts, however, was not different between FX and controls (Figure 6E). These results indicate that FX neuronal networks display a less mature electrical activity pattern compared to WT.

**Figure 7:**
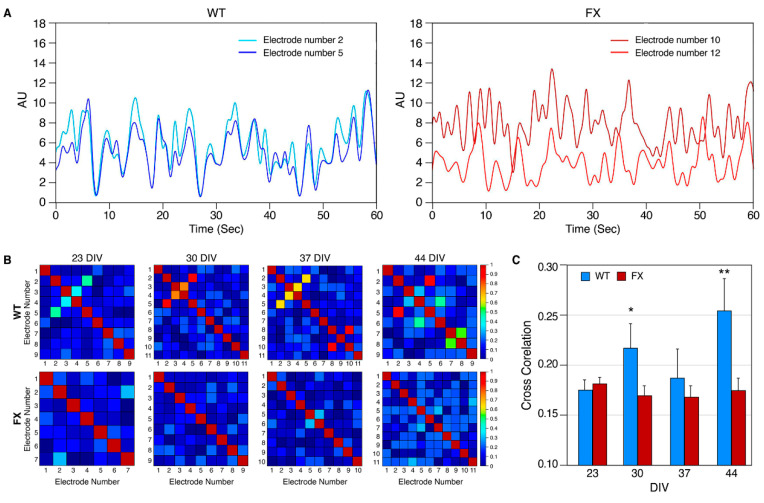
Synchrony of neuronal network activity: (**A**) Representative synchronous (WT, left) and asynchronous (FX, right) electrical activity between two electrodes, (AU—arbitrary unit). The signals shown are spike locations that have been convoluted with Gaussian (sigma = 0.5); (**B**) heat maps summarizing cross-correlation (CC) among all active electrodes (≥15 spikes/min). The panel ranges from high synchrony/high CC among neurons (red), to low synchrony/ low CC (blue). Diagonal red patterns represent autocorrelation within the recorded neurons; (**C**) averaged values of CC electrodes. The threshold was determined to be >0.1. The same batch of neurons was recorded for all time points. Two independent experiments were performed at each time point (23, 30,37 and 44 DIV). A total of 35–207 pairs of electrodes were analyzed for each line in each time point. Data are represented as mean ± SD. *T*-test: * *p* < 0.05; ** *p* < 0.005.

To further compare the functional connectivity between FXS and WT networks, a cross-correlation (CC) analysis was performed (Figure 7). CC measures the distance in time between two spikes generated by different neurons in the same well. If the spikes of one neuron tend to occur at the same time as the spikes of another neuron, the CC score will be close to 1 (red square in the heat map), indicating synchronous network activity. However, if the spikes generated by different neurons constructing the network occur at different time points from the network, activity is considered non-synchronized, and the CC score will be closer to zero (blue square in the heat map of Figure 7B). We analyzed the CC of both FXS and control iNs at four time points during development (23, 30, 37 and 44 DIV). The results show that although both FX and WT neurons mature in culture and their network activity becomes more synchronous (higher CC), in FX, the neuronal network is significantly less synchronous than that of control (0.17 vs. 0.25, respectively, at 44 DIV; *p* < 0.004; Figure 7C).

## 3. Discussion

In the present study we generated isogenic hESC subclones of full-mutation FX- and control-lines that share the same genetic background, and comprehensively compared the functional properties of their derived neurons. We found that FX-iNs that inactivate FMRP show delayed development and structural deficiencies, including significant smaller soma and less-branching neurites. Patch-clamp recordings demonstrated that both FX and WT neurons are able to discharge repetitive APs, and FX neuronal networks are also able to generate spontaneous excitatory synaptic currents with slight differences from WT. These results demonstrate that, by forced expression of *NEUROG-1*, we generate more mature neuronal networks and at a significantly shorter delay than those generated by the dual SMAD inhibition protocol [26]. These iNs represent the neurons that constitute the cerebral cortex as shown also by their expression of *FOXG1*, *BRN2* [22]. The high-throughput MEA system enabled us to explore these differences in a higher resolution also in the network levels, demonstrating that FX neuronal networks are hyperexcitable with a significant higher spontaneous burst-firing activity compared to WT. Most importantly, the quantification of neuronal network synchronicity demonstrates that the FX neuronal networks are significantly less synchronous than the controls.

Isogenic pluripotent stem cell (PSC) lines are currently used for exploring the role of a specific mutation in an underlying disease [27,28,29]. In most studies, isogenic PSC lines are generated through the intervention of genetic manipulation (CRISPR/Cas9), which is commonly associated with off target effects [19,20,25], necessitating their subsequent mutation screening throughout the whole genome before drawing any conclusions. In the current study, we generated isogenic lines, from a hESC line, that carry the natural genotype of FXS with no need for genetic intervention, so that we did not have to suspect off-target effects. The pluripotent Lis_FX6 hESC line used in this study to generate the isogenic clones was derived from an affected blastocyst that presented CGG repeats from the normal through the full mutation range, a native genotype that is also known from FXS patients. Moreover, the advantage of studying FX on hESC rather than iPSC is especially important when analyzing the developmental context of the disease [30].

Forced expression of *NEUROG-1* in hESC, already resulted in neurons a few days following induction as also shown by [22,31]. Neuronal genes were expressed in both WT and FX-iNs, demonstrating that a loss of FMRP does not affect the differentiation potential of hESC into iNs. The FX-iNs express significantly more *GRIA4*, of which its protein, GluR4, is the AMPA receptor subunit of glutamate. GluR4 is highly expressed in the early postnatal hippocampus and is thought to be involved in synaptic plasticity [32,33,34]. The significant higher expression of *GRIA4* in the FX neurons can hint at their immature development compared to the WT cells. In addition, both WT and FX-iNs express FMRP at the pluripotent stage, which is silenced only in full mutation cells following differentiation, mimicking *FMR1* silencing in developing human fetuses [24]. Electrical activity can already be recorded by day 28, compared to >60 days when the dual SMAD inhibition protocol was used [16]. Collectively these results show that our isogenic-derived iNs can serve as a good human in vitro model for exploring the pathophysiology underlying the aberrant cortical function of FXS patients.

A critical feature of neuronal connectivity is manifested in their morphology. Several morphological abnormalities have been described in mice, postmortem human FX neurons [35,36], and in FX-hPSC [16,17,37]. In the present study, we show that n absence of FMRP can already cause a decrease in soma size in iNs at day 7, a phenotype that, in neurons derived by the dual SMAD differentiation protocol, was observed only at day 60. Moreover, smaller cell bodies were shown to reflect neuronal maturation [38] and were also observed in the cortical neurons of ASD-hPSC [39]. Neurites of FX-iNs were longer than WT at early neuronal development, which may indicate a lack of an appropriate synaptic pruning in early development of FX neurons, a process that is required for proper efficient neural communication [40,41,42]. Another feature which may reflect functionality is branching complexity of the neurons. Mature FX-iNs demonstrated significantly less branching, in accordance with what was recently found [17], indicating altered network communication among FX neurons. Several studies already show that astrocytes also have critical effects on neuronal development and neurite growth [43,44], but this has to be further elucidated.

Using patch-clamp recording, together with MEA, we were able to show that FX neurons have increased spontaneous activity, similar to their hyperexcitability previously shown in FX-KO mice [1,45,46], and FX-human cortical neurons [18,19,20,47]. Our in-depth analysis of the MEA results enabled us to show, for the first time, that human FX cortical neurons also display hyper-spontaneous burst-firing activity, with greater frequency and with more spikes in each burst, compared to the control. Hyper-burst activity has been previously suggested in the early development of rodents’ neuronal networks [48,49], which reinforces the suggestion that FX neurons are less mature than the WT.

Most importantly, in the current study, we were able to quantify the synchrony of neuronal network activity by comparing spike trains from a population of FX- and control-networks. By analyzing the interaction between paired spike trains comprising the network, we show that FX neuronal networks are significantly less synchronous compared to the control. Previous studies on FX-KO mice have shown abnormalities in neuronal connectivity and a sharp decrease in the correlated firing of cortical neurons [46,50,51,52]. The synchronization of spontaneous network activity within the cortex is critical for information transfer and is an indicator of functional maturation of the neuronal network. It also plays a major role in the plasticity and development of neuronal circuitry [38,52,53,54].

Disorders characterized by impaired cognitive functions and a deficit of integration of information can result from aberrant integrative circuits that are critical for normal computations [55,56]. Many of the brain’s functions associated with FX, including hyperactivity, hypersensitivity [57] and seizures [58], can be explained by neuronal hyperexcitability and the altered functional connectivity found here in the FXS neuronal network.

Together, these results highlight the value of FXS-hESC-derived iNs for analyzing the difference in excitability between FXS and WT neurons. The higher firing rate and spontaneous synaptic currents, together with the higher spontaneous burst-firing activity, altered functional connectivity and the morphological differences between FXS and WT cells shown here indicate that these are essential attributes of FXS. Obviously, the cells grown in the dish lack systemic effects that influence the neuronal network function *in-vivo*, and thus constitute an excellent system for modeling disease in a dish. Additional functional studies are needed to elucidate the mechanistic role for FMRP in the processes, including neurotransmission and synaptic plasticity, which are thought to be a primary deficit in FXS. By developing such a unique multi-tiered human in vitro approach to tackle FXS, we also provide a preclinical drug screening model, as well as a foundation for studies into other neurodevelopmental diseases with similar poorly characterized etiology and therapy, such as epilepsy and autism.

## 4. Material and Methods

### 4.1. Human Embryonic Stem Cell Culture

The use of spare in vitro fertilization (IVF) embryos, following preimplantation genetic diagnosis (PGD) for the generation of human embryonic stem cells (hESC), was approved by the Israeli National Ethics Committee (7/04-043) and in accordance with the guidelines of the Bioethics Advisory Committee of the Israel Academy of Sciences and Humanities. Lis_FX6 hESC (male; early passage <55), was generated in our lab and shown to carry the full mutation of the *FMR1* gene [23,26]. hESC were cultured on Geltrex (Cat No. A1413202, Thermo Fisher Scientific, Waltham, MA, USA)-coated plates in mTeSR1 medium (Cat No. 85850, STEMCELL Technologies, Vancouver, BC, Canada) supplemented with 100 μg/mL Primocin (Cat No. #ant-pm-1, InvivoGen, San Diego, CA, USA). hESC were passaged using Accutase (Cat No. SCR005, Merck, Darmstadt, Germany) and medium was supplemented with 5 μM ROCK inhibitor Y-27632 (Cat No. 1683, Axon Medchem, Reston, VA, USA) for 24 h to increase survival. hESCs were frozen using CryoStem (Cat No. 05-710-1E Biological Industries, Cromwell, CT, USA).

### 4.2. Single-Cell Cloning

Subclones of full mutation and isogenic control hESCs were derived from Lis_FX6, which was previously shown to carry between 180–500 CGG repeats, as well as some cells that are in the normal range (<50 CGG repeats) [26]. Lis_FX6 hESCs were dissociated into single cells counted and plated into individual Geltrex-coated 96-well plates (10 cells/well). Single isolated colonies were further grown, until established clones were generated, and analyzed for the number of *FMR1* CGG repeats, as well as for their genotypes.

### 4.3. Repeat-Number Assay

The number of *FMR1* CGG repeats was determined using a PCR-based protocol [59] with minor modifications. The protocol combines gene-specific primers that flank the CGG repeats region of *FMR1* gene (Forward: TCAGCTCCGTTTCGGTTTCACTT, Reverse: AAGTACCTTGTAGAAAGCGCCATTG), a polymerase mixture, and a reaction buffer that is optimized for amplification of GC-rich DNA. Since the region flanking the repeats in the PCR product consists of a total of 245 bases, the repeat number associated with each PCR product was derived by using the following formula: *n* ~ (fragment size − 245)/3.

### 4.4. Generation of Induced Neurons from hESC

Doxycycline (Dox)-inducible cells were created following infection of hESC with two lentiviruses: (1) the constitutive expression of the Dox-regulated system, reverse tetracycline transactivator (rTA3) vector; (2) tetracycline-inducible expression of *NEUROG-1* driven by a Tet-Responsive Element (TRE) promoter (a kind gift from Dr. Busskamp, who has previously been shown to successfully generate iNs [60]). Briefly, 5 days before differentiation, an antibiotic selection period was conducted to eliminate non-infected cells with Blasticydin (5 mg/mL) for *NEUROG-1*, and with Hygromycin (50 mg/mL) for rtTA. hESC colonies were dissociated into single cells in Accutase (3 min) and 2 × 10^4^ cells were plated in Neurobasal medium (Cat No. 21103049, Thermo Fisher Scientific) supplemented with 1% Glutamax, 1% NEAA, 2% B27 (Cat No. 17504044, Thermo Fisher Scientific), 2.5% fetal bovine serum (FBS; Cat No. 04-127-1A Biological Industries, CT, USA) and 2 μg/mL Doxycycline (Dox; Sigma-Aldrich #BCBF9827V) onto 13 mm Geltrex coated glass coverslips (Cat No. BNCB00130RA1N, Thermo Fisher Scientific). The medium was changed every other day, and until day 14 following neuronal induction in feeder-free cultures. For the long-term culture of iNs (>14 days), iNs were grown on rat astrocyte as feeders. Dox was kept in the medium until day 7 of differentiation. On days 3–6 Ara-c (Sigma #C1768-100MG) was also added to inhibit hESC proliferation. On day 10, the medium was changed to BrainPhys medium supplemented with 2% SM1 (Cat No. 05792, STEMCELL Technologies) and 10% FBS. After day 10, 50% of the medium in each well was exchanged twice a week.

### 4.5. Rat Astrocytes Feeder Cell Culture

Primary rat cortical astrocytes (Cat No. N7745100 Thermo Fisher Scientific) served as feeders for iN, and were cultured in astrocyte-conditioned medium (ACM) containing DMEM (Cat No. 41965039, Thermo Fisher Scientific) with 10% FBS, 1% N2 Supplement (Cat No. 17502048, Thermo Fisher Scientific) and 1% Pen/Strep (Cat No. 03-033-1B, Biological industries CT, USA). Astrocytes were passaged by Trypsin-B (Cat No. 03-046-1B Biological Industries, CT, USA) 5-7 times before plating to ensure that no rat neurons were present in the culture. Astrocytes were plated (5 × 10^4^ astrocytes in 24-well Geltrex-coated plates), 1–3 days before iN plating.

### 4.6. Immunofluorescence

Cells grown on Geltrex-coated 13 mm glass cover slips were fixed with 4% paraformaldehyde (PFA; Cat No. P6148, Sigma-Aldrich, St. Louis, MO, USA) for 20 min at room temperature (RT). Blocking was performed with blocking solution, 10% FBS with 0.2% Triton X 100 in PBS for permeabilization. Cells were then incubated with primary antibody diluted in blocking solution for 1 h at RT, washed 3 times with PBS and incubated with secondary antibodies for another hour at RT; they were then and counterstained with DAPI for nucleus localization (Cat No. D1306, Thermo Fisher Scientific). Cells were mounted with Fluoromount aqueous mounting medium (Cat No. 00-4958-02, Thermo Fisher Scientific). The antibodies used for the immunostaining are listed in Table 1. Bright-field, phase, and fluorescence images of cells were obtained using an Olympus IX51 inverted light microscope (Japan).

### 4.7. Western Blot Analysis

Protein extraction was performed in ice-cold RIPA lysis buffer containing 1 mM phenylmethylsulfonyl fluoride (PMSF) and a 1% protease inhibitor cocktail (Cat No. p2714, Sigma-Aldrich, MO, USA). Cell lysates were incubated for 20 min on ice, centrifuged and the supernatants were separated on 7.5% SDS-polyacrylamide gel electrophoresis (SDS-PAGE), followed by transfer to nitrocellulose membranes (0.2 μm; Cat No. PB7320, Thermo Fisher Scientific) using a Bio-Rad Mini Trans-Blot Cell. After electrotransfer, the blots were blocked with PBST containing 5% BSA and incubated for 1 h at RT with the primary antibody, the washed with PBST and incubated for 1 h at RT with the secondary antibody. Blots were detected by enhanced chemiluminescence western blotting substrate EZ-ECL (Cat No. RPN2106, Biological Industries, CT, USA), and developed by MYECL Imager (Thermo Fisher Scientific). Western blot analysis was performed on at least three independent biological replicates. The antibodies used for western blot are listed in Table 1.

### 4.8. RNA Extraction and Quantitative Real-Time PCR

Total RNA was extracted using a Direct-zol RNA MiniPrep Kit (Cat No. ZR-R2050 Zymo Research, Irvine, CA, USA), followed by random hexamer-primed reverse transcription using a Superscript IV RT-PCR kit (Cat No. 18091050, Thermo Fisher Scientific). Quantitative real-time PCR (qRT-PCR) was performed using SYBR Green FastMix (Cat No. 95071-012, Quantabio, Beverly, MA, USA). The cycling and analysis were performed using a Rotor Gene 6000 Series (Corbett, QIAGEN, Hilden, Germany) and its complementary analysis software. Standard curves were performed for each gene in every run, and all PCR reactions were performed for three independent experiments with three technical replicates in each experiment. All qRT-PCR assays included a no-template control (NTC) and -RT. *GUSB* served as control to normalize target gene expression. Primer sequences are listed in supporting information, Table 2.

### 4.9. High-Throughput Cell Morphology Analysis

For neurite length and neurite branch point, iNs were plated at a low density (1.5 × 10^4^ neurons/24-well) on rat astrocyte feeder cells. Cultures were fixed and stained with the neuron-specific β-III tubulin marker (*Tuj1*) at four time points (7, 12, 23 and 32 days in vitro; DIV). *Tuj1*-positive iNs were analyzed for neurite morphology using the neurite tracing algorithm (Incucyte imaging system; BioScience, Sartorius, Germany). Frames captured 9–16 separate regions per well using ×20 magnification. Neurite parameters were calculated from a minimum of 40 visual fields per samples, each field containing 40–60 neuronal bodies. The soma perimeter (µm) was measured manually on a bright-field frame (×20 magnification) with CellSens measuring software at each time point (7, 12, 23 and 32 DIV). 

### 4.10. Electrophysiological Recordings

The recordings were conducted as described previously [26]. Action potentials were evoked by injecting depolarizing current pulses. The membrane potential was held at-60 mV in voltage-clamp mode for recording of spontaneous synaptic currents. These were recorded for up to 2 min with a 50 µs sampling rate. Glutamate or GABA were diluted in extracellular recording solution and applied by pressure from an adjacent patch pipette. Signals were amplified with a Multiclamp 700B amplifier and recorded with Clampex 10 software (Axon Instruments, CA, USA). Data were subjected to a 500 Hz low-pass filter and analyzed using Clampfit-10 and Mini Analysis software.

### 4.11. Multielectrode Array (MEAs) Recordings and Data Analysis

Commercial 24-well plate Multielectrode Arrays (MEAs, Multichannel Systems; Reutlingen, Germany), each containing an array of 12 embedded gold electrodes (30 μm in diameter, 300 μm apart), were used to non-invasively monitor the electrical activity of neuronal networks, at different time points, post neuronal induction. The day before cell seeding, MEAs were coated with Geltrex (Cat No. A1413202, Thermo Fisher Scientific) for 2 h in a 37 °C cell-culture incubator. An amount of 2.5 × 10^4^ rat astrocytes was plated in each well one day before the iN seeding; 24 h after the feeder cells were plated, 10^5^ neurons were seeded and cultured in BrainPhys medium supplemented with 2% SM1 (Cat No. 05792, STEMCELL Technologies) and 10% fetal bovine serum (FBS, Cat No. 04-127-1A Biological Industries, CT, USA) in a final volume of 500 μL. The cells were grown in a 37 °C incubator (5% CO_2_ in air), and 50% of the medium was refreshed every 3–4 days. Spontaneous neuronal activity (20 kHz sampling rate; 3 min recording), including firing rate and burst-rate activity were recorded upon mounting the MEA plate into the recording system. The detected spikes were sorted and analyzed using a MATLAB program. The raw data were filtered with a band pass filter between 300–1100 Hz, and the threshold for detecting spikes was determined to be 4.5 times the standard deviation of the average of the entire signal. Only active electrodes, defined as ≥15 spikes /minute (0.25 Hz), were used for analysis. Bursts were defined as ≥3 spikes, with a ≤300 ms interspike-interval on the same burst. In order to investigate synchronization between spike trains in different electrodes in the same well, the Schreiber distance metric was used [61]. By this, each spike was convolved with a Gaussian filter of width σ = 0.5 to form continuous signals, which were then normalized and cross-correlated using the Pearson correlation. The width of the convolving Gaussian filter set the time scale of the interaction between the two spike trains.

### 4.12. Statistical Analysis

Data from different cell lines and treatment conditions were compared using appropriate statistical tests. *p*-values were calculated by paired or unpaired two-tailed Student’s *t*-test or One-way ANOVA using PRISM software; the specific statistical test used is indicated herein.

## Figures and Tables

**Table 1:** Antibodies used for immunofluorescence (IF) and western blot (WB).

Antibody	Experiment (Dilution)	Host	Supplier	Catalog #
OCT4	IF (1:60)	Mouse	Santa-Cruz	sc-5279
FMRP	IF (1:100); WB (1:2000)	Mouse	Biolegend	BLG-834601
Tuj1	IF (1:2000)	Mouse	Biolegend	BLG-801201
Synapsin-I	IF (1:500); WB (1:1000)	Rabbit	Merck	AB1543
GluA2	IF (1:200); WB (1:5000)	Rabbit	Abcam	ab133477
Alexa Fluor 488	IF (1:500)	Rabbit	Thermo Fisher	A11008
Alexa Fluor 488	IF (1:400)	Mouse	Thermo Fisher	A21202
Alexa Fluor 594	IF (1:500)	Rabbit	Thermo Fisher	A11012
β-actin	WB (1:5000)	Mouse	Abcam	ab8226
horseradish peroxidase	WB (1:5000)	Mouse	Cell Signaling Technology	7076s
horseradish peroxidase	(WB 1:5000)	Rabbit	Cell Signaling Technology	7074s

**Table 2:** Primer sequences for qRT-PCR.

Gene	Forward	Reverse
*GUSB*	ATCGATGACATCACCGTCACCA	TCCAAAAGACGCACTTCCAACTTG
*O* *CT* *4*	TTCAGCCAAACGACCATCTG	GGTTCGCTTTCTCTTTCGGG
*TuJ1*	TTT GGA CAT CTC TTC AGG CC	TTT CAC ACT CCT TCC GCA C
*MAP2*	CCTAAGCCATGTGACATCCA	CTGCCTGGGGACTGTGTAAT
*PAX6*	GTGTCCAACGGATGTGTGAG	CTAGCCAGGTTGCGAAGAAC
*FMR1*	TCAAGGGTTGGACCTAATGC	GCAGGAAGCTCTCCCTCTCT
*FOXG1*	TCAGAACTCGCTGGGCAACA	CAGAGCAGGGCACCGACA
*BRN2*	AAAGTAACTGTCAAATGCGCG	GCTGTAGTGGTTAGACGCTG
*SYN1*	CCCCAATCACAAAGAAATGCTC	ATGTCCTGGAAGTCATGCTG
*PSD95*	AGTCAGAAATACCGCTACCAAG	CCGTTCACCTGCAACTCATATC
*GRIA1*	TGATGGAAAATACGGAGCCC	CTTCCCGGACCAAAGTGATAG
*GRIA3*	GTCTTTGGTTTTCCTTGGGTG	CAGCGAGATTGGCAGTATAGG
*GRIA4*	CAAGGAGAGGAAATGCTGGG	ACGTCCATAGTGGTCAAACTG
*GRIK1*	CCTGATGCCTAACACCACATTAACCT	CTGTATGTGTGGAACTTCGAGAGCA
*GRIN1*	GAGAAGGAGAACATCACCGAC	GTC CCCATCCTCATTGAACTC
*mGluR5*	GGTCCAAGAAAAGCAACATCATCAGAT	ATCATCAGTGGGCCAAGACCC
*vGlut2*	TGGTCGTTGGCTATTCTCATAC	ATACTGGCATATCTTGGAGCG
*GABRA2*	CAGCTACTTGCTGAGTGATAGCAAC	CAGGGTCCCACACCAAGAAAAC
